# Low rates of patient-reported outcome claims for orphan drugs approved by the us food and drug administration

**DOI:** 10.1080/20016689.2018.1433426

**Published:** 2018-02-12

**Authors:** Szymon Jarosławski, Pascal Auquier, Borislav Borissov, Claude Dussart, Mondher Toumi

**Affiliations:** a Public Health Department – Research Unit EA 3279, Aix – Marseille University, Marseille, France; b Prescriptia Ltd, Sofia, Bulgaria; c Laboratoire Parcours Santé Systémique EA 4129, Claude Bernard University, Lyon, France; d Faculté de Médecine Laennec, Université de Lyon, Lyon, France

**Keywords:** Patient-reported outcomes, quality of life, rare diseases, orphan drugs, labelling

## Abstract

**Background**: Claims included in package inserts (PIs) for medicinal products approved by the US Food and Drug Administration (FDA) constitute the regulatory definition of drugs’ benefits and risks.

**Objective**: We sought to assess the usage of patient-reported outcome (PRO) claims in a comprehensive set of US FDA orphan drug approvals dated between 1/1/2012 and 31/12/2016, and characterize them.

**Study design**: Orphan drug approval documentation was obtained from the US FDA website. Drug Package Inserts (PI) were analyzed to extract information on PRO-related language.

**Results**: Among 178 drugs that met inclusion criteria, 16 (9%) products approved for 16 orphan indications contained PRO language in the Clinical Studies section of the PI. All PRO instruments concerned disease symptoms, and two also referred to patient functioning. The most common PRO instrument was a bleed-specific rating scale for four products approved for the treatment or prevention of bleeding episodes in patients with genetic bleeding disorders.

**Conclusions**: There is a need to implement public incentives for academic development of PRO instruments for rare conditions and for regulatory policies that mandate the collection of PRO endpoints in pivotal trials of orphan drugs.

## Introduction

The content of package inserts (PIs) for medicinal products approved by the US Food and Drug Administration (FDA) constitutes the regulatory definition of drugs’ benefits and risks. PIs, also called product labels, require FDA approval, define the scope of marketing and can be essential to the commercial success of a medicinal product [1].

According to the FDA, a patient-reported outcome (PRO) is ‘any report of the status of a patient’s health condition that comes directly from the patient without interpretation of the patient’s response by a clinician or anyone else.’ It can be measured in absolute terms (e.g., the severity of a sign, symptom, or state of a disease) or as a change from a previous measure [2]. The PROs encompass but are not limited to measures of Health-Related Quality of Life (HRQoL). A PRO instrument, i.e. a questionnaire plus the information and documentation that support its use, provides means to collect data about a PRO concept [3]. PRO use is more frequent in clinical trials of products for chronic, disabling conditions where the goal of treatment is not curative, but rather to improve symptoms, functioning, or quality of life [4].

The FDA’s guidance on the use of PRO claims in product labelling clarifies that such claims can appear in either the Indications and Usage or Clinical Studies sections of the PI labelling or, more rarely, in its other sections [5]. Further, PRO instrument evaluation principles are the same, regardless of the PI section where the PRO claim appears.

Orphan drugs are products developed to treat rare medical conditions, generally referred to as ‘orphan diseases’. The name ‘homeless or orphan drugs’ was first used in 1968 in the US by G.P. Provost to describe medications in which the pharmaceutical industry seemed to have very little interest [6]. In the US, orphan drugs are formally defined in the Orphan Drug Act as products ‘used in diseases or circumstances which occur so infrequently in the USA, that there is no reasonable expectation that the cost of developing and making available in the USA a drug for such disease or condition will be recovered from sales in the USA for such drugs’ [7]. The prevalence limit for a rare condition is defined as 200,000 people in the US population. In order to accelerate patients’ access to these drugs, less evidence is required for orphan drug approvals compared with non-orphan drugs. Therefore, orphan drug manufacturers may lack the incentive to collect additional data, such as PROs, in the drug development process. Indeed, PROs are rarely reported in clinical trials of drugs for rare diseases [4,8]. Here, we sought to identify and characterise PRO claims in a comprehensive set of FDA orphan drug approvals from a 5-year period.

## Methods

A list of orphan drugs approved by the FDA between 1/1/2012 and 31/12/2016 was obtained from the FDA website. The following products were excluded: vaccines, imaging-related products, products approved based on non-human pivotal trials, products approved based on biosimilarity studies whose PI did not contain the Clinical Studies section, products which most recent PI did not include an orphan indication. The Indication and Usage and the Clinical Studies sections of the PI were reviewed for PRO-related language. PRO claims were classified as measures of symptoms, functioning, HRQoL, patient global rating, or ‘other.’ Further information collected was: PRO instruments named in the PI, PRO endpoint status (primary, secondary, tertiary/exploratory), the statistical significance of the PRO results reported (yes/no), and the pivotal trial design. Descriptive statistics were performed in Microsoft Excel 2016.

## Results

195 orphan-designated drugs were approved by the FDA in the study period, of which 17 products were excluded based on the aforementioned criteria. In terms of therapeutic areas represented in the final sample of 178 products, Oncology had 82 (46%) approvals, followed by Hematology (24, 13%) and Endocrinology (21, 12%). Among products with PRO claims, there were 6 products in Hematology, 4 in Neurology, 2 in Respiratory, and 1 in each of the following areas: Cardiology, Endocrinology, Rheumatology and Other categories.

In total, 16 (9%) products, approved for 16 orphan indications, contained PRO language in the Clinical Studies section of the PI (Table 1). No drugs contained PRO claims in the Indication and Usage section. PIs of three drugs contained two PROs. For 15 of the 16 products (94%), PRO-related results were statistically significant.

**Table 1.** Orphan drugs with PRO labelling.

Generic Name	Indication	Therapeutic area	No. of PROs	PRO tools named in the PI	PRO claim wording in the Clinical Studies section of the PI	PRO endpoint status	PRO result statistically significant	Pivotal trial design
droxidopa	Treatment of neurogenic symptomatic orthostatic hypotension in patients with primary autonomic failure, dopamine-beta-hydroxylase deficiency, and nondiabetic autonomic neuropathy.	Cardiology	1	Orthostatic Hypotension Questionnaire (OHQ)	Efficacy was measured using the Orthostatic Hypotension Questionnaire (OHQ), a patient-reported outcome that measures symptoms of nOH and their impact on the patient’s ability to perform daily activities that require standing and walking. The OHQ includes OHSA Item #1 as one of several components. In one study Efficacy was measured using the OHSA Item #1 score (‘dizziness, lightheadedness, feeling faint, and feeling like you might black out’) at Week 1, in patients who had completed titration and 1 week of maintenance therapy. [Only Item #1 was statistically significant.]	primary	yes	randomised double-blind
tasimelteon	Non-24-hour sleepwake disorder in blind individuals without light perception	Endocrinology	1	Sleep diary	Study 1 and Study 2 evaluated the duration and timing of nighttime sleep and daytime naps via patient-recorded diaries.	primary	yes	randomised double-blind
human factor X	Treatment of hereditary factor X deficiency	Hematology	1	A bleed-specific ordinal rating scale of excellent, good, poor and non-assessable.	The efficacy of COAGADEX in treating bleeding episodes was assessed by the subject and/or investigator for each new bleeding episode, using a bleed-specific ordinal rating scale of excellent, good, poor and non-assessable.	primary	yes	non-randomised open label
C1-esterase inhibitor (recombinant)	Treatment of (acute attacks of) angioedema caused by hereditary or acquired C1-esterase inhibitor deficiency.	Hematology	2	Treatment Effect Questionnaire (TEQ) VAS	PRO 1 pivotal trial: the primary efficacy endpoint was the time to beginning of relief of symptoms, assessed using patient-reported responses to two questions from a Treatment Effect Questionnaire (TEQ). The TEQ required patients to assess the severity of their attack symptoms at each affected anatomic location, using a seven-point scale (‘much worse’ to ‘much better’ [TEQ Question 1]), and whether their symptoms had begun to decrease notably since receiving the study medication (‘yes’ or ‘no’ [TEQ Question 2]). To achieve the primary endpoint, a patient had to have a positive response to both questions along with persistence of improvement at the next assessment time (i.e., the same or better response). PRO 2 trial (used in two separate supportive RCTS): Patients scored their symptoms using a visual analog scale (VAS) ranging from 0-100mm. A VAS decrease of >20 mm compared with baseline with persistence of the improvement at two consecutive time points was considered the onset of relief in Studies 2 and 3. In both Study 2 and 3, the efficacy of RUCONEST in the treatment of acute angioedema attacks was demonstrated by significantly shorter times to beginning of relief of symptoms based on the VAS	primary	yes	randomised double-blind
antihemophilic factor (recombinant), Fc fusion protein	Treatment of hemophilia A	Hematology	1	A 4-point rating scale of excellent, good, moderate, and no response	On-demand Treatment and Control of Bleeding Episodes: Assessment of response to each injection was recorded by subjects at 8–12 hours after treatment. A 4-point rating scale of excellent, good, moderate, and no response was used to assess response.	primary	yes	non-randomised open label
coagulation factor IX (recombinant), Fc fusion protein	Control and prevention of hemorrhagic episodes in patients with haemophilia B (congenital factor IX deficiency or Christmas disease)	Hematology	1	A 4-point rating scale of excellent, good, moderate, and no response	Assessment of response to each injection was recorded by subjects at 8–12 hours after treatment. Excellent: abrupt pain relief and/or improvement in signs of bleeding; Good: definite pain relief and/or improvement in signs of bleeding but possibly requiring another injection in 1–2 days; Moderate: probable or slight beneficial effect and requiring more than one injection; No response: no improvement, or worsening.	primary	yes	non-randomised open label
ecallantide	Treatment of angioedema	Hematology	2	Mean Symptom Complex Severity (MSCS) score Treatment Outcome Score (TOS)	In both trials, the effects of KALBITOR were evaluated using the Mean Symptom Complex Severity (MSCS) score and the Treatment Outcome Score (TOS). These endpoints evaluated attack severity (MSCS) and patient response to treatment (TOS) for an acute HAE attack. MSCS score is a point-in-time measure of symptom severity. At baseline, and post-dosing at 4 hours and 24 hours, patients rated the severity of each affected symptom on a categorical scale (0 = normal, 1 = mild, 2 = moderate, 3 = severe). Patient-reported severity was based on each patient’s assessment of symptom impact on their ability to perform routine activities. Ratings were averaged to obtain the MSCS score. The endpoint was reported as the change in MSCS score from baseline. A decrease in MSCS score reflected an improvement in symptom severity; the maximum possible change toward improvement was −3. TOS is a measure of symptom response to treatment. At 4 hours and 24 hours post-dosing, patient assessment of response for each anatomic site of attack involvement was recorded on a categorical scale (significant improvement [100], improvement [50], same [0], worsening [−50], significant worsening [−100]). The response at each anatomic site was weighted by baseline severity and then the weighted scores across all involved sites were averaged to calculate the TOS. A TOS value >0 reflected an improvement in symptoms from baseline. The maximum possible score was +100.	primary	yes	randomised double-blind
anti-inhibitor coagulant complex	Routine prophylaxis to prevent or reduce the frequency of bleeding episodes in hemophilia A and B patients with inhibitors	Hematology	1	A scale of effective, partially effective, not effective or not sure	Subjects and investigators were asked to rate hemostatic efficacy based on a scale of effective, partially effective, not effective or not sure. The criteria for evaluation of the effectiveness were severity of pain, subjective improvement, circumference of muscle or joint, restriction of joint mobility, cessation of open bleeding, start of rebleeding and quantity and nature of analgesics.	primary	yes	randomised double-blind
dichlorphenamide	Treatment of primary periodic paralyses	Neurology	1	Average number of self-reported attacks of muscle weakness per week	Study 1 was a 9-week, double blind, placebo-controlled multi-center study. Study 1 consisted of two substudies: a substudy in patients with hypokalemic periodic paralysis (n = 44), and a substudy in patients with hyperkalemic periodic paralysis (n = 21). The primary efficacy endpoint in both substudies was the average number of self-reported attacks of muscle weakness per week over the final 8 weeks of the trial. Withdrawal from the study for acute severe worsening was also assessed as an endpoint.	primary	yes	randomised double-blind
levodopa and carbidopa	Treatment of late stage Parkinson’s disease	Neurology	1	Parkinson’s disease diary	The clinical outcome measure in Study 1 was the mean change from baseline to Week 12 in the total daily mean ‘Off’ time, based on a Parkinson’s disease diary. The ‘Off’ time was normalized to a 16-hour awake period, based on a typical person’s waking day and the daily infusion duration of 16 hours. The mean score decrease (i.e., improvement) in ‘Off’ time from baseline to Week 12 for DUOPA was significantly greater (p = 0.0015) than for oral immediate-release carbidopa-levodopa. Additionally, the mean score increase (i.e., improvement) in ‘On’ time without troublesome dyskinesia from baseline to Week 12 was significantly greater (p = 0.0059) for DUOPA than for oral immediate-release carbidopa-levodopa.	primary	yes	randomised double-blind
immune globulin infusion (human)	Treatment of multifocal motor neuropathy	Neurology	2	Guy’s Neurological Disability Scores (GNDS)	Co-primary endpoint: Guy’s Neurological Disability Scores (GNDS) for the upper limbs, reflecting both fine motor skills and proximal strength, showed a significant difference in efficacy between GAMMAGARD LIQUID and placebo at the 2.5% level in favor of GAMMAGARD LIQUID. GNDS is a patient orientated clinical disability scale designed for multiple sclerosis and is considered appropriate for other neurological disorders. Secondary endpoint: Compared to baseline, patients’ assessment visual analog scale (VAS), showed a mean change of 290% during placebo compared to baseline. Patient’s assessments of physical functioning showed a mean change of 73% during GAMMAGARD LIQUID treatment. Higher visual analog scale scores represent more severe disability.	co-primary secondary	yes	randomised double-blind
gabapentin enacarbil	Treatment of postherpetic neuralgia	Neurology	1	Pain Intensity Numerical Rating Scale (PI-NRS)	To ensure that patients had significant pain, randomized patients were required to have a minimum baseline 24-hour average Pain Intensity Numerical Rating Scale (PI-NRS) intensity score of at least 4.0 on the 11-point numerical PI-NRS, ranging from 0 (‘no pain’) to 10 (‘pain as bad as you can imagine’). Treatment with HORIZANT statistically significantly improved the mean pain score and increased the proportion of patients with at least a 50% reduction in pain score from baseline at all doses tested.	primary	yes	randomised double-blind
collagenase clostridium histolyticum	Treatment of Peyronie’s disease	Other	1	Peyronie’s Disease Questionnaire Bother domain score	Peyronie’s Disease Questionnaire Bother domain score (co-primary endpoint): XIAFLEX significantly reduced patient-reported bother associated with Peyronie’s disease compared with placebo.	primary	yes	randomised double-blind
lumacaftor/ivacaftor	Treatment of cystic fibrosis	Respiratory	1	Cystic Fibrosis Questionnaire-Revised (CFQ-R) Respiratory Domain	Key secondary efficacy variables included […] absolute change from baseline in Cystic Fibrosis Questionnaire Revised (CFQ-R) Respiratory Domain score at Week 24, a measure of respiratory symptoms relevant to patients with CF such as cough, sputum production, and difficulty breathing […].	secondary	no	randomised double-blind
ivacaftor	Treatment of patients with cystic fibrosis	Respiratory	1	Cystic Fibrosis Questionnaire-Revised (CFQ-R) Respiratory Domain	Key secondary efficacy variables included absolute change from baseline in Cystic Fibrosis Questionnaire Revised (CFQ-R) Respiratory Domain score at Week 24 and 48, a measure of respiratory symptoms relevant to patients with CF such as cough, sputum production, and difficulty breathing (…).	secondary	yes	randomised double-blind
anakinra	Treatment of cryopyrin-associated periodic syndromes	Rheumatology	1	Disease-specific Diary Symptom Sum Score	NOMID: symptoms were assessed with a disease-specific Diary Symptom Sum Score (DSSS), which included the prominent disease symptoms fever, rash, joint pain, vomiting, and headache.	primary	yes	non-randomised open-label

Abbreviations: CF-Cystic Fibrosis; CFQ-R-Cystic Fibrosis Questionnaire-Revised; DSSS-Diary Symptom Sum Score; GNDS-Guy’s Neurological Disability Scores; MSCS-Mean Symptom Complex Severity; MSCS-Mean Symptom Complex Severity; nOH-neurogenic symptomatic orthostatic hypotension; OHQ-Orthostatic Hypotension Questionnaire; PI-NRS-Pain Intensity Numerical Rating Scale; PI-Package Insert; PRO-Patient Reported Outcome; TEQ-Treatment Effect Questionnaire; TOS-Treatment Outcome Score; VAS-Visual Analogue Scale.

PIs of four products contained PRO results from non-randomised open-label trials and 12 from randomised double blinded trials. All PRO instruments concerned disease symptoms, and two also referred to patient functioning. PROs represented the primary trial endpoint for 14 products (86%) and a secondary endpoint for one product with statistically significant PRO label claims. One product had two PRO claims, one as the primary and one as the secondary endpoint. One further product had a claim of a statistically insignificant PRO as a secondary endpoint.

The most common PRO instrument was a bleed-specific rating scale for four products approved for the treatment or prevention of bleeding episodes in patients with genetic bleeding disorders. The second most common PRO instrument was the Cystic Fibrosis Questionnaire-Revised (CFQ-R) Respiratory Domain for products approved for the treatment of cystic fibrosis. This domain reflects some of the disease symptoms, but not HRQoL or patient functioning.

## Discussion and conclusions

Only 9% of orphan drugs in the studied sample contained PRO claims in their PIs. In contrast, two studies by Gnanasakthy, et al. reported that PIs of 24 and 16.5% of drugs approved by the FDA in 2006–2010 and 2011–2015, respectively, included PRO claims [4,9]. The decreasing trend in the use of PRO claims was explained in part as a result of an increase in oncology product approvals from 15.5% to 27.5% in the respective drug samples. According to the authors, oncology products may have study designs that do not support PRO labelling in compliance with the FDA’s guidance [9]. However, rather than the design of oncology trials per se, the toxicity of oncology treatments can make it challenging to distinguish disease symptoms from treatment side effects in oncology trials, and therefore to demonstrate improvement on PRO endpoints. Nevertheless, given that in our orphan drugs sample oncology products constituted 46%, the low rate of PRO claims could be explained, at least partially, by the high prevalence of cancer products. Further, another study reported a 12% rate of PRO claims in a small sample of orphan drugs, concluding that PRO claims may not be needed for orphan product market differentiation to the same degree as for non-orphan products [10]. Indeed, for many rare disease indications, there are no available therapies and the first one that enters the market will enjoy a monopoly position. Therefore, a PRO claim may not be required for commercial success of such products.

Another explanation for the low rates of PRO claims for orphan drugs is the low number of dedicated PRO instruments available. Since few PRO tools that are specific to rare diseases have been developed, the opportunities to include a PRO claim in the product label are limited [11]. Indeed, the small patient population is an obstacle in recruiting patients to studies designed to develop or validate a PRO tool for a rare disease [12]. Further, rare diseases attract little academic research because it can be more costly and time-consuming, and result in lower citation rates than research on common diseases [13]. Consequently, scientists may be reluctant to pursue research in this field.

Among products with PRO claims included in the label, PROs were the primary trial endpoint in 86%, which is slightly higher than 71 and 76.7% reported previously for all new FDA approvals [4,9]. Much like in the earlier studies [4,9], in our sample all products with PRO claims featured instruments that measured disease symptoms, while the PIs of only two products also included measures of patient functioning. The reluctance of the industry to employ PROs that also measure patients’ functioning and HRQoL in clinical trials can result from the perceived risk of not achieving significant drug efficacy based on such endpoints. This disinclination can be justified in case of drugs that are developed specifically to reduce disease symptoms, such as those reducing the severity of bleeding episodes. However, similar to the studies cited above [4,9], our study did not assess if PROs were collected in clinical trials of the assessed dugs but only if PRO claims were granted by the FDA. This aspect deserves further research.

Generic PRO tools can be less sensitive than disease-specific tools, so that the lack of PRO instruments that are specific to rare diseases can further discourage the industry from incorporating PRO endpoints in clinical trials. However, PROs have the potential to measure not only the symptoms that patients experience, but also their impact on patient’s functioning and HRQoL. This potential of PROs remains underutilised in the area of orphan drugs. Therefore, there is a need to develop new PRO tools that reflect the impact of drugs on multiple domains of patients’ experience, such as functional performance and HRQoL, and to enhance their collection in clinical trials of orphan drugs.

It has been suggested that low rates of PRO collection in clinical trials are underpinned by the lack of regulatory policies that mandate the inclusion of PRO endpoints in drug approval documentation [9]. Nevertheless, given that the FDA launched the Patient-Focused Drug Development initiative in 2013, it may seem that the agency has a strong focus on the patient perspective. For instance, the 2014 Public Meeting on Pulmonary Arterial Hypertension Patient-Focused Drug Development held by the FDA explored patient reported symptoms and their impact on daily life [14]. Surprisingly, whereas the clinical trial of riociguat in patients with pulmonary arterial hypertension included three PRO measures as secondary outcomes (ClinicalTrials.gov Identifier: NCT00810693), none of them was mentioned in the PI of the drug approved in 2013 [15]. Further research is needed to elucidate the FDA’s motivation to omit PRO claims in PIs of products that featured PRO data in their clinical trials.

Given the high burden of developing new PROs for rare diseases, it is unlikely that the low number of PRO claims in this area will increase without suitable policy changes. Therefore, there is a need to implement both public incentives for academic development of PRO instruments for rare conditions and regulatory policies that mandate the collection of PRO endpoints in the pivotal trials of orphan drugs. For instance, such policies could mandate inclusion of secondary PRO endpoints, if they are not used as primary endpoints.
